# Supplementary material for: Enhancing physical activity in older type 2 diabetic adults through remote patient monitoring: a pre-post study in Taiwan
Source: PeerJ. 2025 Jul 9;13:e19659. doi: 10.7717/peerj.19659 (PMC12255238; doi:10.7717/peerj.19659)
Supplement: Supplemental Information 2 [file peerj-13-19659-s002.docx]

| non-English text used in the original raw data file | translation |
| --- | --- |
| 步速：<1m/s為異常 | Gait speed <1 m/s |
| MMSE, Illiterate+Literate≤13/Primary school+Junior high school+Senior high school+University≤24為異常 | MMSE, Illiterate+Literate≤13/Primary school+Junior high school+Senior high school+University≤24 |
| GDS≥2為異常 | GDS≥2 |
| FRAIL分數_Robust 0 Pre-frailty 1或2 Frailty 3以上 | FRAIL score: Robust = 0, Pre-frailty = 1 or 2, Frailty = 3 or above |
