# Supplementary material for: Enhancing physical activity in older type 2 diabetic adults through remote patient monitoring: a pre-post study in Taiwan
Source: PeerJ. 2025 Jul 9;13:e19659. doi: 10.7717/peerj.19659 (PMC12255238; doi:10.7717/peerj.19659)
Supplement: Supplemental Information 3 [file peerj-13-19659-s003.docx]

| **Paper Section/**  **Topic** | **Item No** | **Descriptor** | **Reported?** | |
| --- | --- | --- | --- | --- |
|  |  |  | 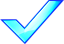 | **Pg #** |
| **Title and Abstract** | | | | |
| Title and Abstract | 1 | 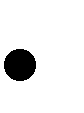 Information on how unit were allocated to interventions | 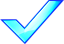 | Line 1 to 4 |
|  |  | 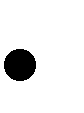 Structured abstract recommended | 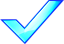 | Line 33 to 83 |
|  |  | 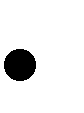 Information on target population or study sample | 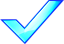 | Line 42 to 43 and line 58 |
| **Introduction** | | | | |
| Background | 2 | 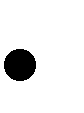 Scientific background and explanation of rationale | 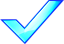 | Line 88 to 120 |
|  |  | 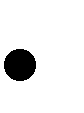 Theories used in designing behavioral interventions | 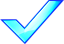 | Line 121 to 148 |
| **Methods** | | | | |
| Participants | 3 | 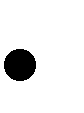 Eligibility criteria for participants, including criteria at different levels in recruitment/sampling plan (e.g., cities, clinics, subjects) | 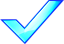 | Line 178 to 187 |
|  |  | Method of recruitment (e.g., referral, self-selection), including the sampling method if a systematic sampling plan was implemented 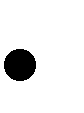 | 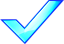 | Line 179 to 180 |
|  |  | Recruitment setting 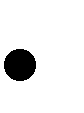 | 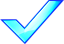 | Line 171 |
|  |  | Settings and locations where the data were collected 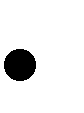 | 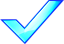 | Line 221 to 223 |
| Interventions | 4 | 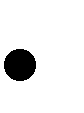 Details of the interventions intended for each study condition and how and when they were actually administered, specifically including: | 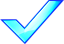 | Line 190 to 216 |
|  |  | - Content: what was given? | 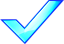 | Line 205 to 206 |
|  |  | - Delivery method: how was the content given? | 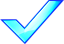 | Line 211 to 215 |
|  |  | - Unit of delivery: how were the subjects grouped during delivery? |  | Line 207 to 208 |
|  |  | - Deliverer: who delivered the intervention? | 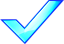 | Line 205 to 206 |
|  |  | - Setting: where was the intervention delivered? | 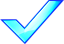 | Line 207 to 208 |
|  |  | - Exposure quantity and duration: how many sessions or episodes or events were intended to be delivered? How long were they   intended to last? | 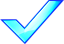 | Line 211 to 213 |
|  |  | - Time span: how long was it intended to take to deliver the   intervention to each unit? | 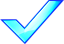 | Line 211 to 213 |
|  |  | - Activities to increase compliance or adherence (e.g., incentives) | 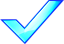 | Line 213 to 216 |
| Objectives | 5 | 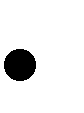 Specific objectives and hypotheses | 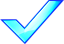 | Line 149 to 159 |
| Outcomes | 6 | 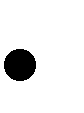 Clearly defined primary and secondary outcome measures | 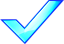 | Line 218 to 221 |
|  |  | 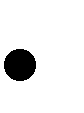 Methods used to collect data and any methods used to enhance the quality of measurements | 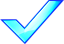 | Line 221 to 223 |
|  |  | 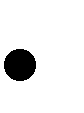 Information on validated instruments such as psychometric and biometric properties | 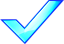 | Line 193 to 197 |
| Sample Size | 7 | How sample size was determined and, when applicable, explanation of any interim analyses and stopping rules 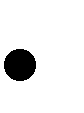 | 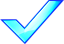 | Line 185 to 187 |
| Assignment Method | 8 | Unit of assignment (the unit being assigned to study condition, e.g., individual, group, community) 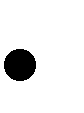 | 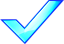 | Line 205 to 206 |
|  |  | Method used to assign units to study conditions, including details of any restriction (e.g., blocking, stratification, minimization) 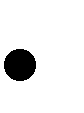 | 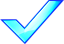 | Line 178 to 185 |
|  |  | Inclusion of aspects employed to help minimize potential bias induced due to non-randomization (e.g., matching) 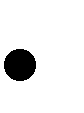 | 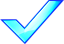 | Line 442 to 454 |

| Blinding (masking) | 9 | 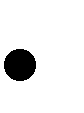 Whether or not participants, those administering the interventions, and those assessing the outcomes were blinded to study condition assignment; if so, statement regarding how the blinding was accomplished and how it was assessed. | 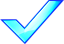 | n/a |
| --- | --- | --- | --- | --- |
| Unit of Analysis | 10 | 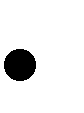 Description of the smallest unit that is being analyzed to assess intervention effects (e.g., individual, group, or community) | 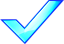 | Line 205 to 206 |
|  |  | 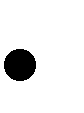 If the unit of analysis differs from the unit of assignment, the analytical method used to account for this (e.g., adjusting the standard error estimates by the design effect or using multilevel analysis) | 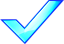 | n/a |
| Statistical Methods | 11 | 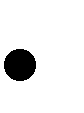 Statistical methods used to compare study groups for primary methods outcome(s), including complex methods of correlated data | 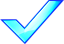 | Line 288 to 292 |
|  |  | 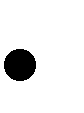 Statistical methods used for additional analyses, such as a subgroup analyses and adjusted analysis | 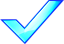 | Line 294 to 303 |
|  |  | Methods for imputing missing data, if used 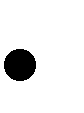 | 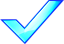 | n/a |
|  |  | Statistical software or programs used 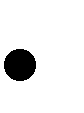 | 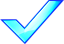 | Line 303 to 305 |
| **Results** | | | | |
| Participant flow | 12 | Flow of participants through each stage of the study: enrollment, assignment, allocation, and intervention exposure, follow-up, analysis (a diagram is strongly recommended) 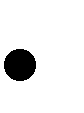 | 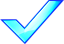 | Line 175 to 176 |
|  |  | - Enrollment: the numbers of participants screened for eligibility, found to be eligible or not eligible, declined to be enrolled, and   enrolled in the study | 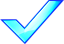 | Line 178 to 180 |
|  |  | - Assignment: the numbers of participants assigned to a study   condition | 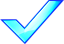 | Line 307 |
|  |  | - Allocation and intervention exposure: the number of participants assigned to each study condition and the number of participants   who received each intervention | 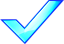 | Line 307 |
|  |  | - Follow-up: the number of participants who completed the follow- up or did not complete the follow-up (i.e., lost to follow-up), by   study condition | 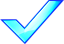 | Line 307 |
|  |  | - Analysis: the number of participants included in or excluded from   the main analysis, by study condition | 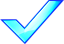 | Line 307 |
|  |  | 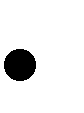 Description of protocol deviations from study as planned, along with reasons | 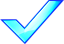 | Line 442 to 453 |
| Recruitment | 13 | Dates defining the periods of recruitment and follow-up 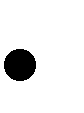 | 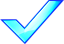 | Line 211 to 215 |
| Baseline Data | 14 | Baseline demographic and clinical characteristics of participants in each study condition 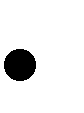 | 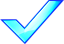 | Line 307 to 321 |
|  |  | Baseline characteristics for each study condition relevant to specific disease prevention research 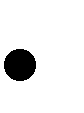 | 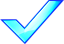 | n/a |
|  |  | Baseline comparisons of those lost to follow-up and those retained, overall and by study condition 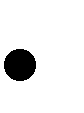 | 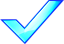 | n/a |
|  |  | Comparison between study population at baseline and target population of interest 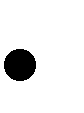 | 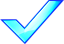 | n/a |
| Baseline equivalence | 15 | 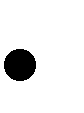 Data on study group equivalence at baseline and statistical methods used to control for baseline differences | 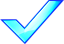 | n/a |

| Numbers analyzed | 16 | 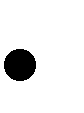 Number of participants (denominator) included in each analysis for each study condition, particularly when the denominators change for different  outcomes; statement of the results in absolute numbers when feasible | 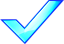 | Line 307 |
| --- | --- | --- | --- | --- |
|  |  | 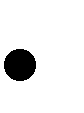 Indication of whether the analysis strategy was “intention to treat” or, if not, description of how non-compliers were treated in the analyses | 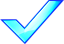 | Yes |
| Outcomes and estimation | 17 | 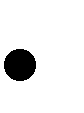 For each primary and secondary outcome, a summary of results for each estimation study condition, and the estimated effect size and a confidence interval to indicate the precision | 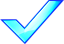 | Line 322 to 336 |
|  |  | 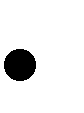 Inclusion of null and negative findings | 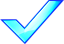 | Line 322 to 344 |
|  |  | Inclusion of results from testing pre-specified causal pathways through which the intervention was intended to operate, if any 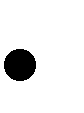 | 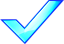 | n/a |
| Ancillary  analyses | 18 | 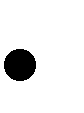 Summary of other analyses performed, including subgroup or restricted analyses, indicating which are pre-specified or exploratory | 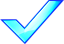 | Line 337 to 344 |
| Adverse events | 19 | 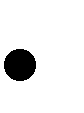 Summary of all important adverse events or unintended effects in each study condition (including summary measures, effect size estimates, and  confidence intervals) | 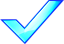 | Line 183 to 187 |
| **DISCUSSION** | | | | |
| Interpretation | 20 | 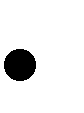 Interpretation of the results, taking into account study hypotheses, sources of potential bias, imprecision of measures, multiplicative analyses,  and other limitations or weaknesses of the study | 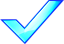 | Line 348 to 457 |
|  |  | Discussion of results taking into account the mechanism by which the intervention was intended to work (causal pathways) or alternative mechanisms or explanations 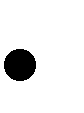 | 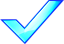 | Line 348 to 457 |
|  |  | Discussion of the success of and barriers to implementing the intervention, fidelity of implementation 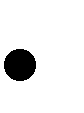 | 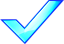 | Line 348 to 457 |
|  |  | Discussion of research, programmatic, or policy implications 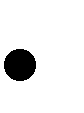 |  | Line 355 to 389 |
| Generalizability | 21 | Generalizability (external validity) of the trial findings, taking into account the study population, the characteristics of the intervention, length of follow-up, incentives, compliance rates, specific sites/settings involved in  the study, and other contextual issues |  | Line 442 to 457 |
| Overall  Evidence | 22 | General interpretation of the results in the context of current evidence and current theory |  | Line 459 to 471 |

*From:* Des Jarlais, D. C., Lyles, C., Crepaz, N., & the Trend Group (2004). Improving the reporting quality of nonrandomized evaluations of behavioral and public health interventions: The TREND statement. *American Journal of Public Health*, 94, 361-366. For more information, visit: <http://www.cdc.gov/trendstatement/>
